# Damaged Hospital Mattresses and Bed Frames Are Common in Acute-Care Hospitals

**DOI:** 10.1017/ash.2021.122

**Published:** 2021-07-29

**Authors:** Edmond Hooker

## Abstract

**Background:** Hospital beds are now high-tech, reprocessable, medical devices. The surface of the mattress (cover) is manufactured using polyurethane-coated fabric to ensure moisture-vapor transmission to prevent pressure ulcers. In recent years, due to multidrug-resistant organisms, healthcare organizations have used increasingly harsh chemicals to clean these mattresses. None of these chemicals are approved for use on polyurethane-coated fabric. Previous research has shown that many mattresses in hospitals are damaged. The US Food and Drug Administration, Centers for Disease Control and Prevention, ECRI, and manufacturers recommend routine mattress inspection and replacement of mattresses with any visible signs of stains, wear, or damage. Damaged mattresses have been linked to fluid leakage, resulting in patient exposure and outbreaks of healthcare-acquired infections. **Methods:** Four hospitals of a midwestern hospital system had all of their mattresses inspected for damage and staining to the mattress. After external examination, each mattress was opened, and the mattress core was evaluated for damage. The cover of each mattress was examined using the naked eye and then using an LED light to demonstrate smaller holes. Each bed frame was examined for evidence of rust, and the amount of rust was recorded. If available, the age of the mattress was determined based on a label on the mattress. **Results:** In total, 727 beds and mattresses were inspected. Of these mattresses, 523 (72%) were damaged. Also, 340 (47%) required replacement of the mattress cover, and 183 (25%) required replacement of the entire mattress (cover and core). For the 209 damaged mattresses (40%) with the date of manufacture label, 156 (75%) were <4 years old. Damage to the mattress included 428 (59%) with holes in the cover: 113 (16%) were visible to the naked eye and 315 (43%) small holes only detected by using an LED light. Also 173 mattresses (24%) had stains on the exterior cover, 215 (30%) had stains on the interior of the top cover, and 192 (26%) had stains on the interior of the bottom cover. Bed-frame rust was identified on 175 (24%) beds, of which 65 (9%) had widespread rust. **Conclusions:** These findings confirm previous reports that damaged mattresses are common in hospitals and potentially place patients at risk. Most of these failed mattresses are <4 years old, which is much less than the expected life of a mattress and bed deck.

**Funding:** No

**Disclosures:** None

